# Delivery-mediated exosomal therapeutics in ischemia–reperfusion injury: advances, mechanisms, and future directions

**DOI:** 10.1186/s40580-024-00423-8

**Published:** 2024-04-30

**Authors:** Shengzhe Ding, Yu-Jin Kim, Kai-Yu Huang, Daniel Um, Youngmee Jung, Hyunjoon Kong

**Affiliations:** 1 Chemical & Biomolecular Engineering, University of Illinois, Urbana, IL 61801 USA; 2 Center for Biomaterials, Korea Institute of Science and Technology, Seoul, 02792 Republic of Korea; 3 Bioengineering, University of Illinois, Urbana, IL 61801 USA; 4 Carl R. Woese Institute for Genomic Biology, University of Illinois, Urbana, IL 61801 USA; 5 Department of Electrical and Electronic Engineering, YU-KIST Institute, Yonsei University, Seoul, 03722 Republic of Korea; 6 Chan Zuckerberg Biohub-Chicago, Chicago, USA; 7 KU-KIST Graduate School of Converging Science and Technology, Korea University, Seoul, 02841 Republic of Korea

## Abstract

Ischemia-reperfusion injury (IRI) poses significant challenges across various organ systems, including the heart, brain, and kidneys. Exosomes have shown great potentials and applications in mitigating IRI-induced cell and tissue damage through modulating inflammatory responses, enhancing angiogenesis, and promoting tissue repair. Despite these advances, a more systematic understanding of exosomes from different sources and their biotransport is critical for optimizing therapeutic efficacy and accelerating the clinical adoption of exosomes for IRI therapies. Therefore, this review article overviews the administration routes of exosomes from different sources, such as mesenchymal stem cells and other somatic cells, in the context of IRI treatment. Furthermore, this article covers how the delivered exosomes modulate molecular pathways of recipient cells, aiding in the prevention of cell death and the promotions of regeneration in IRI models. In the end, this article discusses the ongoing research efforts and propose future research directions of exosome-based therapies.

**Graphical Abstract:**

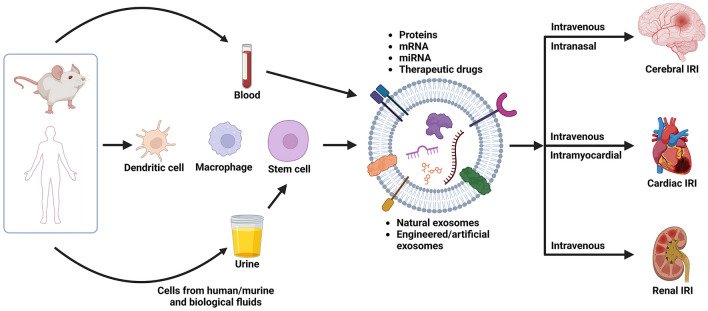

## Introduction

Ischemia-reperfusion injury (IRI) is a complex pathological condition resulting from blood supply suddenly returning to the ischemia site and causing oxidative damage [1]. A series of cellular events are triggered by this abrupt reintroduction of blood, which may exacerbate tissue damage beyond the initial ischemic trauma. This phenomenon occurs in a variety of medical scenarios, ranging from transplant surgeries to strokes and heart attacks, making IRI one of the significant challenges in modern clinical therapies [2, 3].

Current medical approaches to IRI mitigation encompass a range of strategies, each with its own strengths and limitations (Table 1). One of these strategies involves carefully restoring blood flow to the ischemic area, minimizing the hypoperfusion period, and consequently mitigating cellular damage [4, 5]. However, the therapeutic window for such interventions is often narrow, necessitating prompt action to yield optimal results. Another method applies therapeutic hypothermia, an approach that has demonstrated protective effects against ischemic brain injury, but optimal temperature and timing are being debated [6–8]. Besides, pharmacological reagents targeting oxidative stress, such as N-acetylcysteine and N-mercaptopropionylglycine, has shown promise in preventing myocardial IRI [9]. Calcium channel blockers such as verapamil, diltiazem, or nifedipine have also been found to relieve IRI [10]. Furthermore, cyclosporine A has been reported to inhibit excessive opening of mitochondrial permeability transition pores (mPTP), which results in the reduction of ROS generation [11]. However, there is still a need to repair or resuscitate vascular and tissue cells with dysfunctional mitochondria and damaged membranes [3].

**Table 1:** Current therapy and challenges for cerebral, cardiac, and renal IRI

Types	Current therapy	Major challenges
Cerebral IRI	Antioxidants, hypothermia, remote ischemic conditioning	BBB disruption [12], seizures, malignant cerebral edema [13]
Cardiac IRI	Antioxidants, hypothermia, remote ischemic conditioning [14]	Exacerbated and accelerated myocardial injury [15]
Renal IRI	Antioxidants, remote ischemic conditioning [16]	Risk of progression to chronic kidney disease [17]

**Fig. 1:**
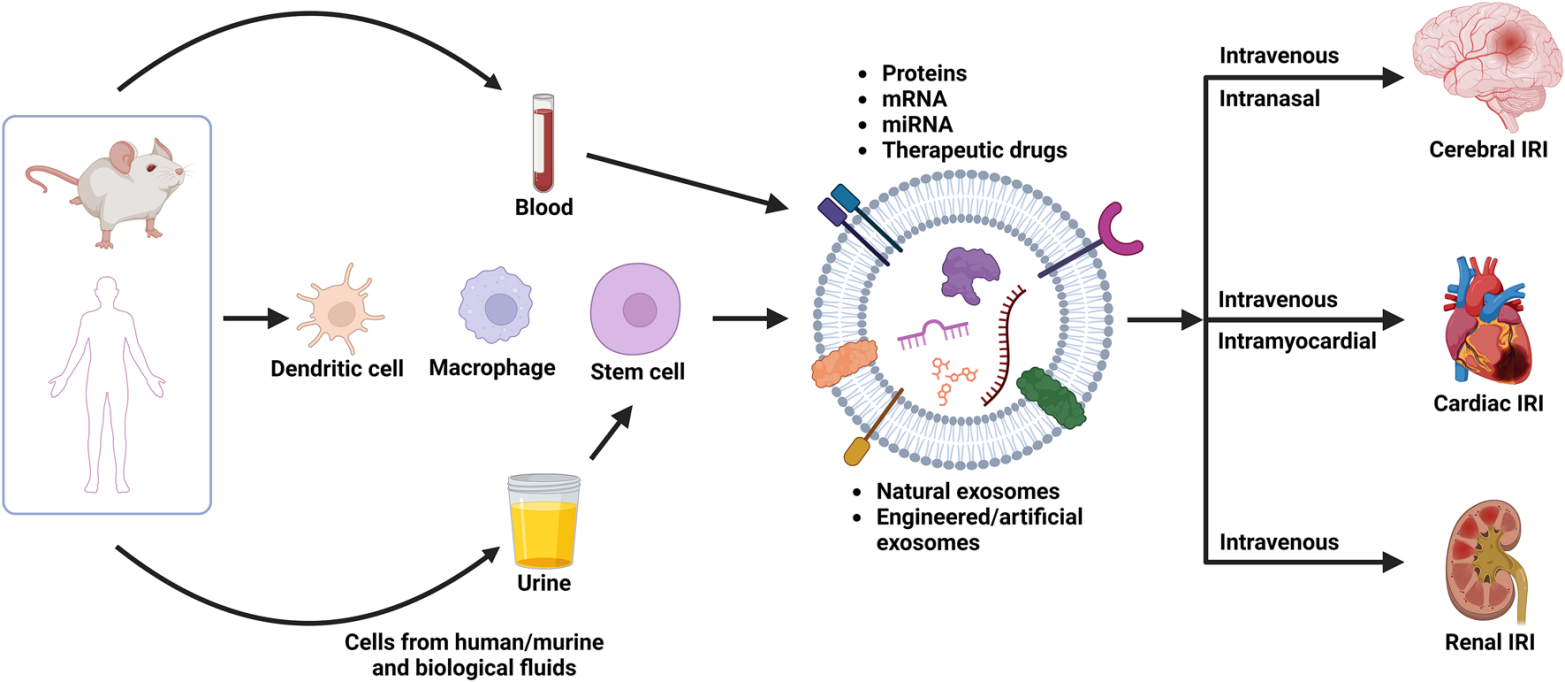
Recent advance in sources of exosomes and therapies for ischemia-reperfusion injury. Exosomes derived from various sources are administered through different routes as a novel therapy for cerebral, cardiac, and renal IRI. Created with BioRender.com

To this end, exosomes are emerging as a new wave of therapeutics to treat this exacerbation of cellular dysfunction and tissue death, as illustrated in Fig. 1. Biological cell-secreting exosomes with diameters ranging from 30 to 150 nm have received lots of attention due to their potential to regulate intercellular communication responsible for tissue homeostasis, repair, regeneration, and inflammation [18, 19]. Exosomes are generated mainly from the reverse budding of multivesicular bodies. Containing a rich content of signaling proteins and lipids inherited from parental cells on the surface, exosomes demonstrate the ability to transport a diverse range of molecules across major biological membranes, including the blood–brain barrier (BBB) [20–22]. These vesicles enter cells and release microRNA and mRNA cargo that can modulate gene expression and translation of the cells. The exosomes, costumed by the originating cell type, undergoes internalization through different delivery mechanism, includes receptor-mediated interactions, membrane fusion, and endocytosis [23]. A few delivery routes have been reported to deliver exosomes. For example, intravenously introduced M2 microglia-derived exosomes improved recovery and motor function in mice with spinal cord injury by inhibiting neurotoxic A1 astrocyte activation and the NF-κB signaling pathway [24]. Also, intranasal delivery of human adipose-derived stem cell exosomes at 48 h post-traumatic brain injury in mice has demonstrated significant recovery in motor and cognitive functions [25]. Prior studies have demonstrated that exosomes derived from sources such as mesenchymal stem cells (MSCs) and macrophages possess the capacity to treat IRI by fostering angiogenesis, diminishing inflammation, and enhancing cell survival. These therapeutic effects are largely attributed to the regulatory functions of miRNAs within the exosomes. In the context of treating IRI, exosomes are typically harvested from stem cells due to their potent regenerative properties. However, the therapeutic efficacy of exosomes is often varied by several factors in exosome sources and delivery route, thus affecting the reproducibility of exosome efficacy [26, 27].

Therefore, this review article seeks to provide an overview of the efficacy and challenges of current exosome-based IRI therapies. In particular, we will discuss the extent to which source of exosome and administration strategy influence the efficacy in treating IRI in the heart, kidney, and brain by promoting revascularization while limiting proinflammation and further cellular damage. This review article provides comprehensive insights into the potential of exosome-based interventions for treating IRI, thus contributing to advancing exosome therapeutics for IRI.

## IRI mechanism

IRI is a medical state caused by abrupt blood flow restoration preceded by temporarily limited blood supply to localized areas of the body [28]. This pathologic state leads to tissue deterioration and degeneration and, ultimately, organ failure [29–32]. This inadvertent pathologic complication results from the interaction of two phases: Ischemia, which is defined by inadequate oxygen and blood delivery, and reperfusion, which is the restoration of blood flow following ischemia [33]. Although essential for tissue survival, reperfusion paradoxically sets off a chain of events that magnifies cellular damage [34, 35].

In the ischemia stage, a sudden sequence of biochemical and metabolic alterations takes place due to the deprivation of oxygen and nutrients [36]. Anaerobic conditions and metabolic acidosis in the ischemic tissue lead to cell damage through cytotoxic effects. These effects include reduced adenosine triphosphate (ATP) production, hindered pH-sensitive enzyme synthesis, mitochondrial impairment, metabolic dysfunction, and apoptosis [1, 37–39]. The following reperfusion therapy aiming to restore blood flow triggers undesired overproduction of reactive oxygen species (ROS). These ROS increase intracellular oxidative stress abnormally, resulting in DNA damage, protein malfunction, and lipid peroxidation [40, 41].

Mitochondria is a key organelle that produces 90% of intracellular ROS. IRI induces mitochondrial hyperpolarization that leads to increased ROS production. These ROS make mitochondria dysfunctional and prompt the discharge of impaired mitochondrial DNA [42–44]. According to the study on molecular patterns associated with mitochondrial damage, ROS activates NLR family pyrin domain-containing 3 (NLRP3) inflammasome [45, 46]. These inflammasome-induced signaling pathways release pro-inflammatory cytokines, including IL-1β and IL-18 [46]. Separately, ROS activates endothelium to overproduce intercellular adhesion molecules (ICAM)-1 and fibrinolytic inhibitors on the vascular wall [47]. Through multiple activations of transcription factors, cytokines, and adhesion molecules, IRI causes severe inflammation, negatively impacting tissue structure and function.

The activated immune system also contributes to IRI. Reperfusion therapy following ischemia injury recruits neutrophils and macrophages rapidly and causes inflammation [48]. In particular, these recruited immune cells release cytokines, chemokines, and proteases, thus aggravating tissue damage. Increased tissue damage from IRI has been related to sterile inflammation [1, 48]. This non-infectious inflammation is also represented by the activation of Toll-like receptors (TLRs) and NOD-like receptors (NLRs) [48]. Also, sterile inflammation has been accompanied by cytokines and chemokines such as TNF- α, IL-1, and CCR2, which is similar to infectious inflammation [49]. Moreover, the complement system, a part of the innate immune response, is activated, adding another layer of complexity to the inflammatory cascade. Double-stranded DNA (dsDNA) from ischemic tissues and mitochondrial dysfunction by IRI leads to the elicitation of cyclic GMP-AMP synthase (cGAS) and stimulator of interferon response cGAMP interactor 1 (STING1) pathway, which leads to the secretion of IL-6, IFNβ1, and TNF [46, 50].

In addition, IRI also results from the disturbance of calcium homeostasis. Ischemia impairs ion pumps necessary to sustain calcium gradients across cellular membranes. Then, reperfusion therapy causes calcium influx that activates the production of cellular damage-inducing enzymes such as phospholipases, proteases, and endonucleases. Excessive intracellular calcium also impacts mitochondria by opening mitochondrial permeability transition pores and causing energy collapse [51]. These understandings necessitate interventions that can regulate multifaceted and complicated IRI pathologies.

Based on these aforementioned mechanisms, IRI is characterized by a sequence of intricate processes that vary across different organs due to their distinct physiological and metabolic characteristics [52]. This condition is marked by a combination of oxidative stress, inflammation, and programmed cell death [53]. In the heart, IRI is precipitated by the restoration of blood flow to previously ischemic heart tissue, setting off a cascade of chemical and cellular reactions [54]. Principal among these is the excessive generation of reactive oxygen species (ROS), an abrupt increase in calcium within cells that disrupts mitochondrial function, and the initiation of pathways that lead to cell death [55]. Additionally, damage to the endothelium and the invasion of neutrophils amplify harm to the cardiac tissue [56]. The kidneys, when subjected to IRI, experience damage mainly in the tubules and blood vessels, leading to a condition known as acute tubular necrosis and a decrease in the filtration rate of the glomeruli [57, 58]. This condition is driven by mechanisms similar to those in the heart, with significant effects resulting from dysfunction of the endothelial cells and death of tubular cells. Furthermore, the kidneys suffer from a distinctive type of harm due to the reverse flow of the filtrate, which aggravates the deterioration of renal performance [59]. Brain tissue is especially susceptible to IRI due to its high requirement for metabolic substrates and its sensitivity to damage from excessive neuronal stimulation [60, 61]. When blood flow is reinstated after an ischemic event, there is an overproduction of glutamate and other neurotransmitters that excessively activate NMDA receptors, causing an influx of calcium, mitochondrial impairment, and neuronal cell death [62–64]. The compromise of the blood–brain barrier also plays a part in this process, leading to swelling in the brain and inflammatory responses [65].

## Exosome-based therapy for cerebral IRI

### Intravascular administration of exosomes

Cerebral IRI, a detrimental consequence following the restoration of blood flow post-ischemic stroke, often results in neuronal damage, worsening the post-stroke recovery [66, 67]. Treatment considerations for cerebral IRI emphasize the need to address the acute and long-term neuroinflammatory responses, minimize neuronal death, and enhance neural repair mechanisms. Recent research has indicated that exosomes can cross the BBB and regulate the brain function [20]. With this unique transport ability, exosomes can mitigate the molecular and cellular events responsible for cerebral IRI (Table 2). Strategically, exosome therapy for the brain involves leveraging their ability to carry neuroprotective agents, anti-inflammatory substances, and molecules that can promote neurogenesis and angiogenesis directly to the site of injury, offering a targeted approach to enhancing post-stroke recovery.

**Table 2:** List of recent exosome-based in vivo studies for cerebral IRI

Exosome source	Modifications	Cargo	Delivery route	Animal model	References
M2 microglia	–	miR-124	Intravenous; tail vein	tMCAO, mouse	[73]
Neutrophil membrane	Resolvin D2 loading	Resolvin D2	Intravenous	tMCAO, mouse	[79]
Macrophage	Heptapeptide loading	Heptapeptide	Intravenous; tail vein	tMCAO, mouse	[80]
Neural progenitor cell	–	–	Intravenous; femoral vein	tMCAO, mouse	[66]
Adipose-derived MSC	–	miR-760-3p	Intranasal	tMCAO, mouse	[78]

Delivering therapeutics to the central nervous system is crucial for treating cerebral IRI, but the BBB poses a significant challenge by restricting the passage of most pharmacological substances. Invasive delivery methods, while effective, are often undesirable due to risks like systemic toxicity and complications [68, 69]. Consequently, exosomes have emerged as a novel, non-invasive delivery vector that can naturally traverse the BBB. Their inherent stability, coupled with their non-immunogenic nature, allows for safe passage through the body’s defense mechanisms [70, 71]. Moreover, the specificity of exosomal cargo targeting offers a precise approach to delivering therapeutic agents directly to affected central nervous system tissues [72].

**Fig. 2:**
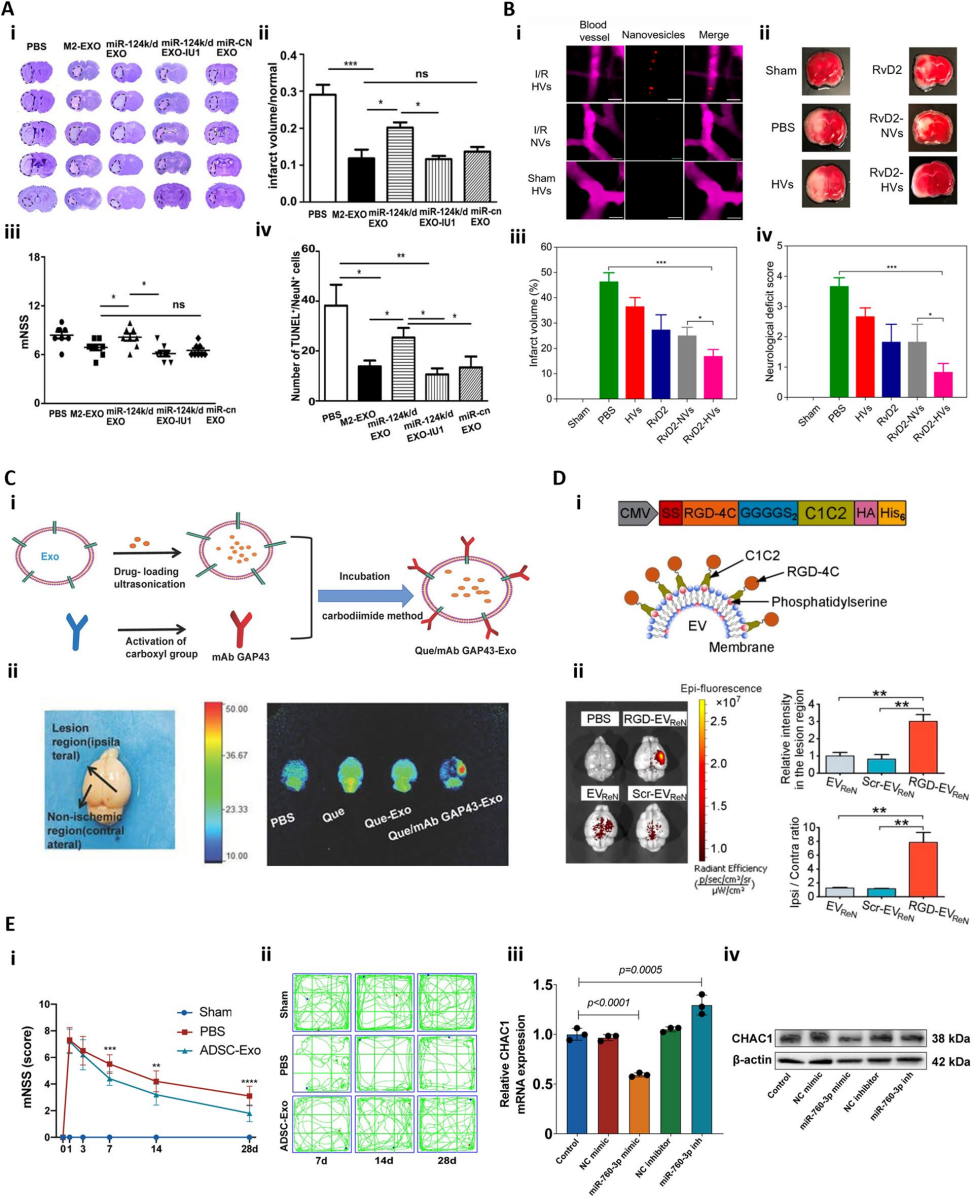
Exosome-based therapy for cerebral IRI. **A** Tail vein injection of M2 microglia-derived exosomes (M2-EXO) attenuated cerebral IRI by downregulating of protease 14 (USP14) expression. (i) Cresyl violet-staining of brain sections, (ii) normalized infarct volume %, (iii) modified neurological severity score (mNSS), and (iv) number of apoptotic neurons in the brain of the mice treated with PBS, M2-EXO, miR-124 k/d EXO, miR-124 k/d EXO + IU1 and miR-cn EXO (exosomes from M2 microglia treated with a control lentivirus vector) [73]. **B** Intravenous injection of RvD2-loaded artificial exosomes, synthesized from the membranes of differentiated human promyelocytic leukemia cells (HL-60), reduced neurological damage following tMCAO. (i) Confocal images showed Dil-labeled nanovesicles (red), from differentiated HL-60 (HVs), adhering to the inflamed brain vasculature post tMCAO while nanovesicles from non-differentiated HL-60 (NVs) showing minimal binding. Blood vessels visualized by BSA-Cy5 (pink). Scale bar = 20 μm. (ii) TTC-stained brain sections revealed (iii) Infarct sizes and (iv) neurological deficit scores at 22 h after injection Reprinted with permission from [79]. Copyright 2019 American Chemical Society. **C** Intravenous injection of mAb GAP43-conjugated exosomes targeted specifically to damage neurons post cerebral IRI. (i) Scheme of conjugating mAb GAP43 to quercetin (Que)-loaded exosome surface to assemble Que/mAb GAP43-Exo. (ii) Fluorescence images of brains from tMCAO rats treated with PBS, Que, Que-Exo, and Que/mAb [81]. **D** Neural progenitor cell-derived exosomes modified with RGD peptides enhanced lesion targeting through intravenous injection. (i) Design of RGD-C1C2 and RGD-C1C2-decorated EV. (ii) Near Infrared Fluorescence images of mice brains the intravenous administration of PBS, Cy5.5-labeled EV, Scr-EV or RGD-EV. Quantitation of fluorescence intensity in the lesion region and ratios of fluorescence intensity in ipsilateral versus contralateral region [76]. **E** Intranasal administration of exosomes derived from adipose-derived MSC (ADSC-Exo) improved neurobehavior function and inhibited ferroptosis by downregulating CHAC1 expression via miR-760-3p. (i) Neurological deficits were evaluated using mNSS before and after tMCAO. (ii) Motor function was assessed. (iii) RT-qPCR results of CHAC1 mRNA expression. (iv) Western blot showing CHAC1 protein expression [82]

A recent study employed M2 microglia-derived exosomes (M2-EXOs) were reported to attenuate cerebral IRI in murine models by miR-124 cargos that downregulated ubiquitin-specific protease 14 (USP14) [73]. Male ICR mice were subjected to 1 h of transient middle cerebral artery occlusion (tMCAO) followed by reperfusion. Post-occlusion, mice received daily injections of M2-EXOs and miR-124 knockdown exosomes (miR-124 k/d EXO), with or without IU1, an inhibitor for USP14, via the tail vein for 3 days (Fig. 2A). The administration of M2-EXOs significantly reduced infarct volume (Fig. 2A–i&ii), enhanced neurobehavioral outcomes as quantified by the modified neurological severity score (Fig. 2A-iii), and inhibited neuronal apoptosis (Fig. 2A-iv). These therapeutic benefits were diminished when miR-124 was knocked down within the exosomes. Intriguingly, co-administering IU1 with miR-124 k/d EXOs showed comparable neuroprotective results to those achieved with M2-EXOs alone, thereby validating the vital role of miR-124/USP14 pathway in protecting brain against IRI.

Separately, an artificial exosome was created by loading Resolvin D2 (RvD2) into neutrophil membrane-derived nanovesicles (Fig. 2B). RvD2, biosynthesized from docosahexaenoic acid, has anti-inflammatory activity with potential as a leukocyte regulator [74]. These nanovesicles were designed to target the inflamed brain vasculature with the presence of integrin β_2_ and P-selectin glycoprotein ligand-1 (PSGL-1) on the vesicle surface and deliver RvD2 to counteract neuroinflammation after IRI in the transient middle cerebral artery occlusion (tMCAO) mouse model. Nanovesicles derived from differentiated human promyelocytic leukemia cells (HV) were administered intravenously after 1 h tMCAO. These engineered exosomes adhered onto the inflamed brain blood vessel due to interaction between integrin β_2_ on the exosome and ICAM-1 on the endothelim (Fig. 2B-i). The administration of HV containing RvD2 significantly reduced tMCAO-induced brain damage, decreasing infarct volume fraction from 46% with no-treatment condition to 16% (Fig. 2B-ii&iii), and diminishing the neurological deficit score (Fig. 2B-iv).

The therapeutic potential of exosomes in the context of cerebral IRI could be further enhanced by implementing surface modifications to improve targeted delivery [75–77]. For example, Growth Associated Protein 43 (GAP43), known to be upregulated in neurons upon injury or stimulation, has been targeted by conjugating monoclonal antibodies (mAb GAP43) to the surface of blood-derived exosomes through carbodiimide-mediated reaction (Fig. 2C-i). These engineered mAb GAP43-conjugated exosomes, when administered intravenously via the tail vein, successfully target the neurons damaged by tMCAO in a rat model (Fig. 2C-ii) [75]. In a parallel approach, exosomes originating from neural progenitor cells were functionalized with integrin-binding peptides, such as RGD peptides, to boost the affinity of exosomes for the lesion site (Fig. 2D). This targeting precision was attributed to the interaction of RGD-modified exosomes with integrin α5β3 with the comparison to scramble (Scr) peptide-decorated exosomes, which is highly expressed in endothelial cells near the lesion region, thereby facilitating the traversal of these engineered exosomes across the blood–brain barrier (BBB) [76].

#### Intranasal administration of exosomes

Intranasal delivery of therapeutic agents has emerged as a promising method to efficiently transport exosomes to the brain through olfactory and trigeminal nerves, bypassing the BBB. A recent study explored the therapeutic potential of adipose-derived MSC exosomes in ischemic stroke [78]. When administered intranasally, the MSC-derived exosomes reached the brain without the need to modify the surface. Similarly, MSC-derived exosomes delivered intranasally into the tMCAO model significantly improved neurobehavioral function (Fig. 2E-i&ii). This therapeutic benefit was attributed to inhibiting neuron-specific ferroptosis, a process associated with cell death after ischemic injury by suppressing the expression of CHAC1 with miR-760-3p (Fig. 2E-iii&iv) [78].

### Exosome-based therapy for cardiac IRI

Cardiac IRI occurs when obstructed blood flow to the heart is restored abruptly. In particular, current acute myocardial infarction (AMI) treatments, such as thrombolytic agent administration and angioplasty, primarily focus on restoring blood flow. However, these procedures sometimes inadvertently exacerbate the reperfusion injury or pose risks like hemorrhagic complications [83]. For cardiac IRI, treatment considerations focus on minimizing the extent of myocardial damage, reducing inflammatory responses, and preserving cardiac function. In this section, we will explore the potential of therapeutic exosomes in cardiac IRI management. Therapeutic exosomes could provide a targeted approach by carrying cardioprotective molecules directly to the heart, aiming to mitigate inflammation, decrease infarct size, and support myocardial repair and regeneration (Table 3).

**Table 3:** List of recent exosome-based in vivo studies for cardiac IRI

Exosome source	Modifications	Cargo	Delivery route	Animal model	References
Bone marrow MSC	–	miR-182-5p	Intravenous	30-min LAD occlusion and reperfusion, mouse	[93]
Human umbilical cord MSC	AT-EHBPE/CP05 peptide hydrogel	Not analyzed	intramyocardial	90-min LAD occlusion, mouse	[109]
Human cardiosphere-derived cell	–	Not analyzed	intracoronary + intramyocardial	90-min LAD occlusion and 30-min reperfusion, pig	[106]
Human cardiosphere	conjugated with cardiac homing peptide	miR21, miR146a	Intravenous	30-min LAD occlusion, mouse	[100]
M2 macrophage	–	Not analyzed	Intravenous; tail vein	30-min LAD occlusion and 2 h reperfusion, rat	[110]
Human neuronal stem cell	–	Not analyzed	Intravenous; jugular vein	40-min LAD occlusion and 2 h reperfusion, mouse	[111]
Cortical bone stem cell	–	miR-378a, let-7a-3, and miR-31	intramuscular	45-min left coronary artery ligation and reperfusion, mouse	[112]
Mouse adipose-derived MSC	–	miR-224-5p	Intravenous; tail vein	30-min left coronary artery (LCA) ligation and reperfusion, mouse	[91]
Myocardial fibroblasts	–	miR-133a	intramyocardial	30-min LCA ligation and 120-min reperfusion, rat	[113]
Remote ischemic preconditioning plasma	–	miR-24	intramyocardial immediately after ligation	45-min LAD occlusion and 24 h reperfusion, rat	[114]
Human plasma	–	miR-486	intraperitoneally after ligation	30-min LAD occlusion and 24 h reperfusion, rat	[115]
Adult rats and human blood	–	HSP70	Intravenous; tail vein	30-min LAD occlusion and 120-min reperfusion, rat	[116]
C57BL/6 mouse MSC	–	miR-21-5p	intramyocardial	45-min LAD occlusion and reperfusion, mouse	[117]
Human embryonic stem cell-derived MSC	–	Not analyzed	Intravenous of CM before reperfusion; tail vein	30-min LCA ligation and 24 h reperfusion, mouse	[98]

#### Intravascular administration of exosomes

Systematic and intracoronary injection is widely adopted for delivering exosomes as therapeutic vehicles because exosomes can pass biological barriers and protect the encapsulated cargo from the immune system and degradation [84]. Additionally, exosomes have been shown to have tropism through different tissues, allowing them to target specific areas [85]. Furthermore, intravascular injection of exosomes has been found to result in their uptake by organs such as the liver, spleen, and lungs [86].

Researchers have recently paid attention to exosomes as potential therapeutic agents for cardiac IRI. These exosomes have been shown to present abilities to reduce apoptosis, augment proliferation, and bolster cardiomyocyte function [87, 88]. For instance, a study studied therapeutic potentials of exosomes from adipose tissue-derived MSCs in alleviating myocardial infarction (MI) symptoms. Microarray analysis revealed that miR-671, alleviating inflammation, [89] was significantly upregulated after exosome treatment in cardiomyocytes, which underwent oxygen–glucose deprivation (OGD). Conversely, downregulation of miR-671 blocked the protective functions of exosomes. The miR-671 targeted transforming growth factor-β receptor-2 and suppressed phosphorylation of Smad2., to enhance the viability of cardiomyocytes undergoing OGD. These findings suggest that miR-671 in MSC-derived exosomes targets and modulates the TGFBR2/Smad2 axis, providing a promising avenue for cardiac IRI therapy [90].

**Fig. 3:**
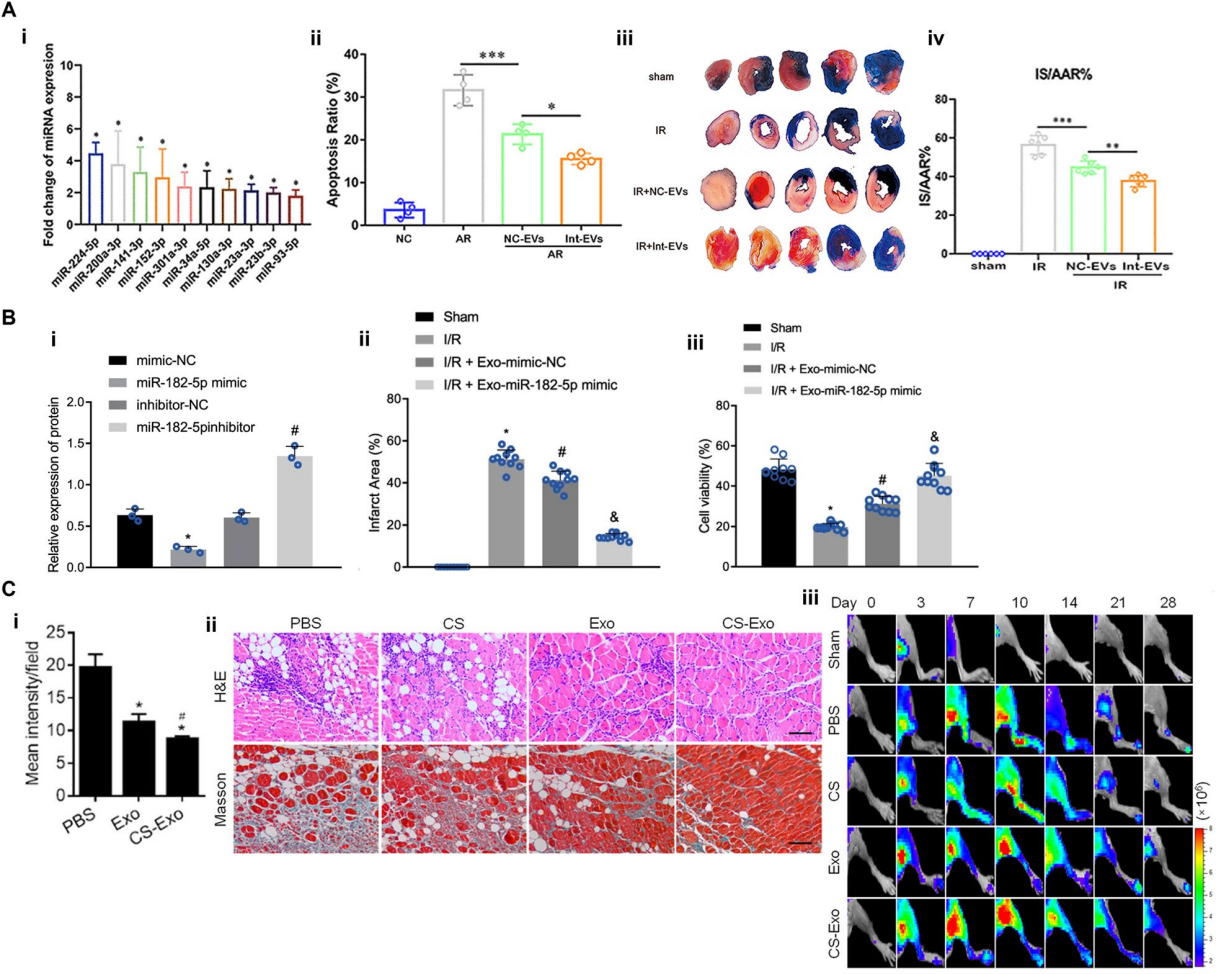
Exosome-based therapy for cardiac IRI. **A** Extracellular vesicles (EVs) produced by adipose-derived MSCs reduce pyroptosis and apoptosis in cardiomyocytes subjected to anoxia/reoxygenation (AR) by downregulation of pyroptosis related gene TXNIP. (i) Validation of differential EV-associated miRNA expression through qRT-PCR. (ii) Apoptosis detection in AR-exposed cardiomyocytes. The nC-EVs group was compared with the AR group and the Int-EVs group was compared with the nC-EVs group. (iii, iv) Assessment of infarct size (IS) and area at risk (AAR) in mice subjected to myocardial ischemia reperfusion injury [91]. **B** MSC-derived exosomal microRNA-182-5p alleviates myocardial ischemia/reperfusion injury by targeting Gasdermin D (GSDMD) in mice. (i) Western blot analysis of GSDMD protein in myocardial cells, normalized to GAPDH. *N* = 10 for mice in each group. (ii) Myocardial infarction (MI) size diagram calculated by the cross-sectional imaging. (iii) The cell viability in myocardial tissues measured by Calcein-AM/PI double staining [93]. **C** Effects of MSC-derived exosomes with an injectable hydrogel for hindlimb ischemia treatment. (i) Quantification of the apoptosis index of HUVECs administrated with exosome (Exo) and chitosan hydrogel to form hydrogel-incorporated Exo (CS-Exo) under H2O2-induced hypoxic stress. (ii) Representative images of muscle sections stained with H&E at day 14 and Masson’s trichrome at day 28 for apoptotic fibers analysis. Scale bar = 100 μm. (iii) In vivo monitoring of the status of angiogenesis following transplantation of CS-Exo or Exo through tracking *Vegfr2-luc* expression by bioluminescence imaging in mouse hindlimb ischemic models. Reprinted with permission from [101]. Copyright 2018 American Chemical Society

Also, extracellular vesicles (EVs) from mouse adipose-derived MSCs, especially when preconditioned under anoxia, exhibit enhanced cardioprotection efficacy in myocardial IRI (Fig. 3A). Anoxia-preconditioned EVs, rich in miRNA224-5p (Fig. 3A-i), were administered intravenously in a mouse IRI model created by left coronary artery ligation and reperfusion. This treatment significantly reduced infarct size and cardiomyocyte apoptosis (Fig. 3A-ii&iii&iv) [91].

Bone marrow MSCs (BMSCs) also release exosomes encapsulating therapeutic microRNAs (miRNAs) that can mediate diverse biological functions. For instance, exosomes containing miR-182-5p were intravenously delivered to a mouse IRI model created by 30-min left anterior descending coronary artery occlusion followed by reperfusion. These exosomes had the potential to alleviate cardiac IRI by targeting Gasdermin D, which is a substrate of caspases 1 and 11. These caspases cleave the gastrin-D to release proinflammatory IL-1β and IL-18, inducing pyroptosis, which is lytic programmed cell death [92]. Exosomes containing miR-182-5p delivered into mouse IRI model also improved cardiac function and diminished myocardial infarction (Fig. 3B) [93]. Moreover, a range of miRNAs, including miR-19a, miR-21, and miR-210, housed within these exosomes displayed cardioprotective effects, promoting cardiomyocyte survival and function while mitigating cardiac fibrosis [94–97]. Notably, several of these miRNAs also induced angiogenesis in post-myocardial infarction ischemic hearts, highlighting the dual therapeutic potential of MSC-derived exosomes in both cardiac regeneration and angiogenesis following cardiac injury.

Moreover, human embryonic stem cell-derived MSCs were shown to secrete exosomes that have cardioprotection activities. Intravenous administration of MSC-conditioned medium before reperfusion significantly reduced infarct size in a mouse model subjected to 30 min left coronary artery ligation followed by 24 h reperfusion. However, additional analysis should be followed to fully identify the constituents of these exosomes [98].

In addition, efforts are made to improve homing of exosomes to target cardiac IRI sites by modifying exosome surface. For instance, cardiosphere-derived exosomes are coated with cardiac homing peptides (Sequence: CSTSMLKAC) as enlighten by a previous study [99]. These modified exosomes were delivered intravenously in a mouse IRI model induced by a 30 min LAD occlusion and reperfusion. Such approach enhanced efficacy of peptide-modified exosomes in promoting cardiac repair, evidenced by reduced scar size, increased cardiac cell proliferation, and enhanced angiogenesis, compared with exosomes coated with a chemically identical but has a randomized internal sequence [100]. 

#### Intramyocardial administration of exosomes

Intramyocardial injection of exosomes was also adopted in treating cardiac IRI with the aim of achieving localized, direct therapeutic effects within the heart tissue. This method bypasses systemic circulation, ensuring a higher concentration of exosomes reaches the desired site of action within the heart [102, 103]. Despite the risk of exosome leakage from the injection site and venous drainage, significant myocardial retention and uptake are still achieved in the crucial initial hours post-injection [104, 105]. This targeted approach is beneficial in scenarios where localized treatment is crucial, such as in cases of acute myocardial infarction or ischemic heart disease, where timely and concentrated exosome delivery can significantly enhance tissue repair and recovery [106].

Exosomes derived from cardiosphere-derived cells were applied to treat myocardial IRI. Intramyocardial injection after 90 min LAD occlusion-induced ischemia and 30-min reperfusion was more effective than intracoronary delivery, as characterized with more significantly decreased infarct size and better retention of left ventricular ejection fraction (LVEF). Magnetic resonance imaging also demonstrated that percutaneous intramyocardial delivery of cardiosphere-derived cell-secreting exosomes preserved left ventricle volumes and LVEF while reducing scar size. The precise mechanism or pathway through which these exosomes exert their therapeutic effects is yet to be investigated [106]. 

Exosomes are also injected into or sprayed onto cardiac lesions resulting from IRI injury. However, the fast loss of exosomes by interstitial fluid flow raises the need for frequent administration of exosomes. To enhance the retention and stability of exosomes, exosomes bound to injectable conductive hydrogel are delivered. Then, the hydrogel provides mechanical support while enabling sustained exosome release. Therefore, this delivery strategy allows one to provide a sufficient therapeutic dose to the lesion area by avoiding repeated intramyocardial injections and prolonging the half-life of exosomes while preventing rapid clearance. For example, bone marrow MSC-derived exosomes encapsulated in a gelatin methacryloyl (GelMA) hydrogel were sprayed onto the surface of the heart with myocardial infarction. This process led to enhanced cardiac function recovery. We envisage that this technique can be easily extended to treating cardiac IRI, particularly during the bypass surgery to reperfuse a heart with myocardial infarction [107].

In addition, we suggest that exosome-encapsulating gel constructs used to treat limb IRI can be adapted to treating cardiac IRI. For instance, intramuscular injection of chitosan hydrogel-loaded human umbilical cord-MSC derived exosomes into a hindlimb ischemia mouse model. This approach enhanced retention and stability of exosomes, reduced muscle cell apoptosis (Fig. 3C-i), and suppressed tissue fibrosis compared to simply injecting exosomes (Fig. 3C-ii), and enhanced angiogenesis (Fig. 3C-iii) [101]. Exosomes encapsulated in a silk fibroin hydrogel also led to the sustained release, reducing aging-induced vascular dysfunction in the mouse hindlimb ischemia mouse model [108].

### Exosome-based therapy for renal IRI

Acute kidney injury (AKI) induced by IRI is a significant contributor to end-stage renal disease [118]. Treatment considerations for renal IRI emphasize the importance of addressing the immediate damage caused by ischemia and the subsequent fibrotic processes that impair renal recovery. Using murine kidney IRI models created by renal pedicle clamping for a certain period and subsequent clamp removal, several studies demonstrated that MSC-derived exosomes could protect renal function. This promising outcome was achieved by mitigating inflammation and fibrosis [83] and enhancing revascularization [119, 120]. A comprehensive list of these and other recent exosome-based in vivo studies for renal IRI can be found in Table 4. In general, delivering drugs to kidneys is challenging due to their rapid blood flow and complex structure [121–123]. In contrast, leveraging exosomes’ cellular or organ tropism holds the potential to specifically target the kidneys, addressing these delivery challenges and promoting more efficient drug delivery to enhance renal protection and repair [121–125].

**Table 4:** List of recent exosome-based in vivo studies for renal IRI

Exosome source	Modifications	Cargo	Delivery route	Animal model	References
Human umbilical cord-derived MSC	N/A	miR-125b-5p	Intravenous at 0 and 24 h of reperfusion; tail vein	30 min both renal pedicle occluded and 48 h reperfusion, mouse	[124]
Human renal tubular cells	N/A	Not analyzed	Intravenous at 24 h of reperfusion; tail vein	50 min both renal pedicle occluded and reperfusion, rat	[129]
Mouse tubular epithelial cell in IR condition	Virus^(CD26) + transfection	CD26	Intravenous; tail vein	IRI model, no detail	[130]
USC	N/A	miR-146a-5p	Intravenous upon reperfusion; dorsal vein of the penis	45 min left renal pedicle and reperfusion, right kidney removed; rat	[133]
Human embryonic kidney cells (HEK293)	EXPLOR technology	exosomal super-repressor inhibitor of NF-ĸB (Exo-srIĸB)	Intravenous	IRI model, no detail	[134]
Human umbilical cord-derived MSC	N/A	Not analyzed	Intravenous	120 min left renal artery ligation and reperfusion, right kidney removed, pig	[125]

**Fig. 4:**
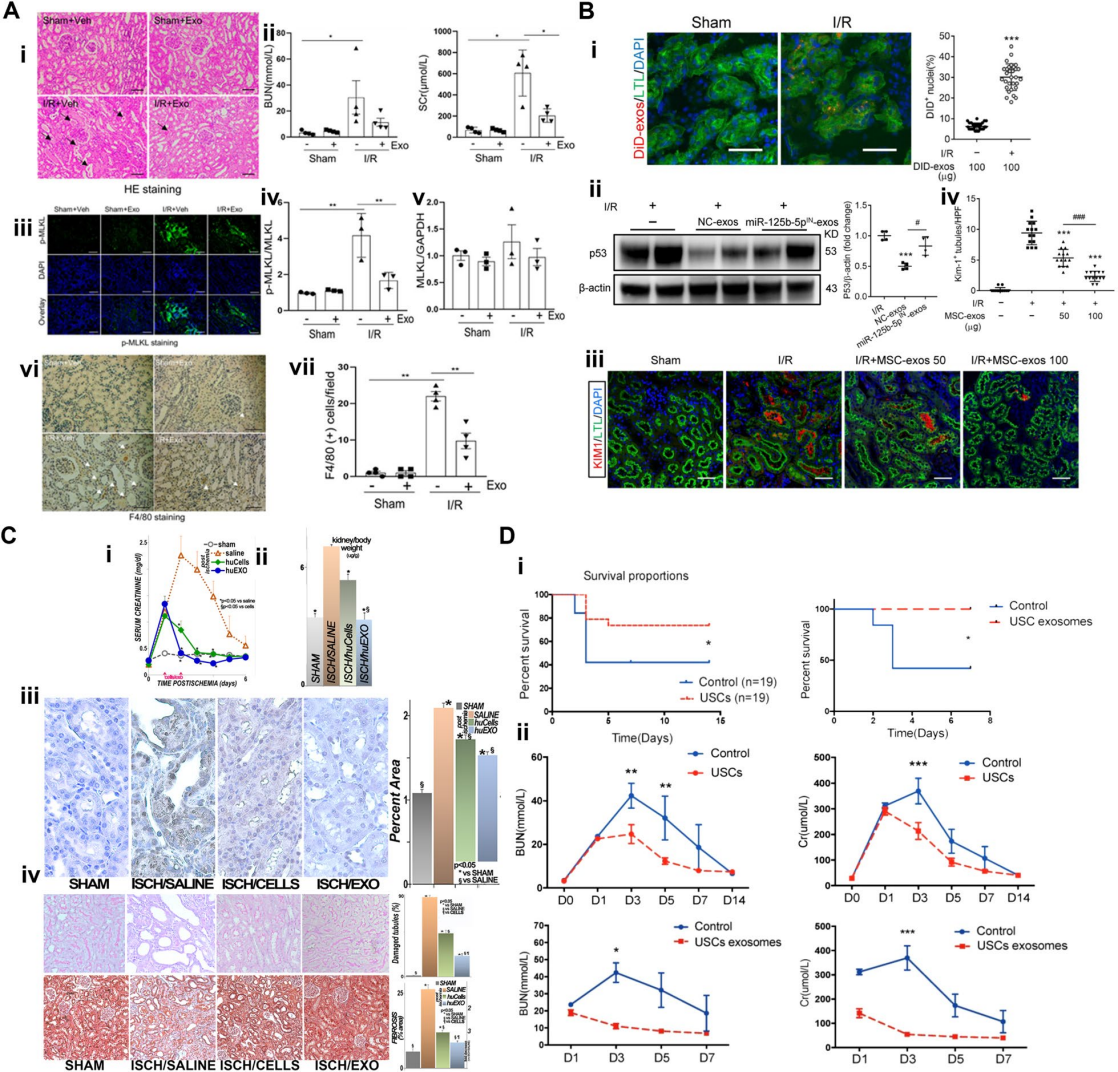
Exosome-based therapy for renal IRI. **A** The renal IRI in miniature pigs was significantly reduced at 72 h after treatment. (i) Arrows point to injured tubules with intraluminal casts with H&E staining.. Scale bar = 100 µm. (ii) Serum creatinine (SCr) and BUN were significantly reduced, indicating improved renal function. (iii, iv, & v) IRI-induced expression and phosphorylation of MLKL were suppressed by exosomes. Scale bar = 200 µm. (vi) Arrows point to F4/80-positive macrophages. Scale bar = 100 µm. (vii) Counts of F4/80 positive cells [125]. **B** MSC-derived exosomes accumulated in renal tubules of IRI mice, mitigating damage by rescuing G2/M arrest and reducing apoptosis via inhibition of p53 signaling through miR-125b-5p. (i) DiD-labeled MSC-exos accumulated in renal tubules of renal IRI mice. (ii) Western blot of p53 in NC-exos or miR-125b-5p^IN^-exos treated IRI mice. (iii) Confocal images of KIM-1 + positive tubules. (iv) Quantification of KIM-1 + tubules per high power field. Scale bars, 25 μm [124]. **C** Human primary renal tubular cell and exosome therapy mitigated rat renal IRI. (i) Serum creatinine levels. Sham (sham), untreated (saline), IRI rats treated with human kidney cells (huCells) and human exosomes (huEXO). At 48 h post-IRI, creatine level of huEXO group was significantly lower than huCells group. (ii) Kidney weights reduced after cell and exosome treatment. (iii) Oxidative stress, indicated by 4-hydroxynonenal adducts (brown), was substantially decreased following exosome therapy. (iv) Cellular attenuation and tubular dilation were improved in ISCH/CELLS and largely prevented in ISCH/EXO [129]. **D** Survival and renal function improved in rat IRI models treated with USC and USC-derived exosomes. (i) Survival rates significantly increased for both USC and USC-exosome groups. (ii) Decreased BUN and creatinine (Cr) levels indicated improved renal function [133]

Certain studies have indicated that intravenously injected human umbilical cord MSC-derived exosomes accumulate in injured proximal tubules and are taken up by renal proximal tubular epithelial cells in IRI-induced AKI mouse models [126]. This homing event was also observed in a miniature pig, where intravascularly injected exosomes accumulated in kidneys affected by IRI-induced AKI (Fig. 4A-i) [125]. The targeting delivery was proposed to be facilitated through integrins, a major component of exosomes [124, 127, 128]. Fundamentally, tubular epithelial cells, fibroblasts, and podocytes in the kidney exhibit elevated expression of distinct integrin subtypes. These integrins are crucial in regulating cell adhesion and intracellular signaling [127]. IRI-induced expression and phosphorylation of pseudo-kinase mixed lineage kinase domain-like protein (MLKL) was suppressed by exosomes as detected by immunoblot analysis (Fig. 4A-iii, iv, &v). These exosomes further promoted renal regeneration and function, as shown by the reduced serum creatinine and blood urea nitrogen (BUN) level (Fig. 4A-ii) and enhanced protective factors such as Klotho and bone morphogenetic protein-7 expression. They also attenuated inflammation by inhibiting pro-inflammatory cytokines and macrophage infiltration (Fig. 4A-vi&vii). Additionally, MSC-derived exosomes preserved renal angiogenesis by promoting expression of VEGFA, VEGFR2, and CD31 [125].

This targeting ability is attributed to the surface markers very late antigen (VLA)-4, integrin α4β_1_, and lymphocyte function-associated antigen (LFA)-1 on the exosome surface as these integrins interact with vascular cell adhesion molecule 1 (VCAM-1) and intercellular adhesion molecule-1 (ICAM-1), facilitating the homing of MSC-derived exosomes to inflamed kidneys where VCAM-1 and ICAM-1 expression is elevated during renal IRI. In a dose-dependent manner, exosomes intravenously administrated through the tail vein were shown to accumulate in renal tubules in IRI mice (Fig. 4B-i), significantly alleviate murine ischemic AKI, decrease renal tubular injury, reduce cell cycle arrest, and reduce apoptosis of tubular epithelial cells. These reparative effects are mechanistically linked to the miR-125b-5p present in exosomes, which downregulates p53 in tubular epithelial cells (Fig. 4B-ii). This interaction enhanced cell cycle progression and reduced apoptosis, ultimately promoting tubular repair. This was shown by the downregulation KIM-1 expression by MSC-exos treatment in a dose-dependent manner (Fig. 4B-iii&iv). Throughout this process, exosomes transmitted miR-125b-5p to tubular epithelial cells, reducing G2/M arrest and inhibiting apoptosis, thereby promoting tubular repair in the mouse IRI model [124]. 

Macrophage-derived exosomes were also capable of reaching the injured kidney through integrin interactions. Notably, these exosomes were found to be more internalized in proximal tubular epithelial cells that were particularly sensitive to ischemic injury. Even when IL-10 was loaded within exosomes, there was no difference in the exosomes’ ability to target injured kidneys. These exosomes also contributed to renal recovery in ischemic injury by inhibiting mTOR signaling, inducing mitophagy to maintain mitochondrial fitness, and increasing polarization towards M2 macrophages [128].

In addition, human renal tubular cell derived exosomes collected from culture medium by sequential centrifugation demonstrated promising activities to mitigate kidney IRI in rats. Intravenous administration at 24 and 48 h post-reperfusion through the tail vein, exosomes conserved both renal function and structure better than the saline control group as shown by the serum creatinine levels (Fig. 4C-i). Lowered kidney weights suggested reduced organ swelling with cell and exosome treatment (Fig. 4C-ii). The exosomes conferred resistance against oxidative stress (Fig. 4C-iii) and curtailed apoptosis while dampening inflammatory and fibrogenic pathways. Histological analysis revealed the therapeutic potential of exosomes through alleviating cellular attenuation and tubular dilation (Fig. 4C-iv). Notably, proteomic analyses revealed that exosome treatment favorably rectified 377 protein profiles out of the 628 renal proteins altered by ischemic injury [129]. CD26-enriched exosomes from tubular epithelial cells were also shown to enhance renal recovery post-renal IRI by fostering cell proliferation and mitigating inflammation. Adenovirus expressing mouse CD26 was utilized to transfect the tubular epithelial cells and produce CD26-enriched exosomes. These intravenously injected exosomes dampened the expression of the cell cycle inhibitors p53 and p21, thereby aiding tissue repair. Furthermore, they reduced inflammatory infiltration by downregulating chemokine receptor CXCR4 and stromal derived factor-1 (SDF1) in the kidney, contributing to the alleviation of renal injury [130]. 

Urine-derived stem cells (USCs) recently emerged as a promising source of exosomes that can treat renal IRI. These stem cells can proliferate up to 10 passages in vitro and secrete exosomes with the potential to prevent and mitigate kidney injury by inhibiting apoptosis and promoting vascular regeneration [131, 132]. These USC-derived exosomes are rich in miR-146a-5p and target and downregulate interleukin 1 receptor-associated kinase (IRAK)-1, a gene implicated in inflammatory responses. This interaction inhibits NF-κB signaling, reducing renal inflammation and cell apoptosis. The injection of USCs and USC-derived exosomes increases the survival rate of rats with IRI (Fig. 4D-i). The USC-derived exosomes intravenously delivered through the dorsal vein of the penis more effectively decreased serum creatinine and blood urea nitrogen levels (Fig. 4C-ii), mitigating renal injury in the rat IRI model field than the intravenously delivered USCs [133]. 

More interestingly, the USC-derived exosomes exhibited better efficacy than USC transplantation. Following injection of stem cells into the rats via the dorsal vein of the penis, most stem cells were found to be trapped in lung tissue rather than the damaged kidney tissue, as confirmed with human nuclear antigen staining performed at day 7 post-injection. Few USCs were integrated into the tubular epithelial lining of the kidney. In short, USC-derived exosomes present a promising and non-invasive strategy for treating renal IRI, circumventing the limitations of direct stem cell transplantation [133].

Additionally, A study administrated an exosomal super-repressor inhibitor of NF-κB (Exo-srIκB) to mice undergoing renal IRI surgery. This treatment effectively decreased markers of kidney damage including blood urea nitrogen, creatinine, and neutrophil gelatinase-associated lipocalin, outperforming the control group receiving exosomes directly from human embryonic kidney cells HEK293T. Furthermore, Exo-srIκB was shown to suppress NF-κB signaling and apoptosis in the affected kidneys while reducing the expression of pro-inflammatory cytokines and adhesion molecules [134].

## Conclusion and future perspectives

### Conclusion

This review has explored the growing field of exosome-based therapies in the treatment of IRI in the brain, heart, and kidney. Across the spectrum of cardiac, cerebral, and renal IRIs, exosomes have demonstrated promising therapeutic potentials with their innate abilities to target tissue, modulate inflammatory response, suppress oxidative stress, enhance angiogenesis, and promote tissue repair. Importantly, the effectiveness of exosome therapy is modulated by biotransport routes and cellular sources, thus raising the need to carefully determine administration strategies and characterize exosome cargos based on IRI-induced pathogenesis. Furthermore, as noted, a deeper understanding of the transport mechanisms of exosomes into cells will be crucial in maturing their therapeutic applications for IRI treatments, enhancing the potential of exosomes as a transformative approach in regenerative medicine and IRI management. In this regard, we noted a few future perspectives below.

### Future perspectives

#### Mechanistic understanding on efficacy of exosomes

This mechanistic understanding involves the exploring of exosomal content in cellular communication and repair. Understanding how these exosome cargos are altered by the cell of origin or disease state is crucial for utilizing exosomes with enhanced therapeutic efficacy. In addition, recent studies put efforts to modify exosome surfaces for targeted delivery with tissue targeting peptide [135, 136]. These approaches improved stability and circulation time against the reticuloendothelial system or the mononuclear phagocyte system [22, 137–142]. Furthermore, among specific subpopulations of exosomes, the promise of exosome-based therapies for IRI is significantly enhanced by the expression of CD47 in which aids in the evasion from the mononuclear phagocyte system, thereby improving their stability and circulation time when delivered intravascularly [143, 144]. This specificity reduces off-target effects and increases the therapeutic potential of exosomal IRI therapies. Future research on exosomal IRI therapies can adopt these modifications in the aspect of stability, transport, and biodistributions [145].

#### Clinical translation and standardization

Bridging the gap between laboratory research and clinical application is essential for the success of exosome-based IRI therapies. This includes conducting clinical trials to validate the safety and efficacy of exosome therapies and determining the most effective methods of administration for IRI in different tissues [146]. Despite ongoing clinical trials exploring the use of exosomes for various treatments, there are no trials specifically investigating their application in treating IRI. Furthermore, the standardization of exosome manufacturing including production, isolation, and storage is vital. To increase natural exosome production, many strategies have been adopted including inducing hypoxia [147], increasing the volume of cell culture from flasks to containers, bioreactors, or hollow fibers [148, 149], and reversing quiescence and prevent senescence of MSCs [150]. In this context, efforts for reliable extraction and purification of exosomes are being made by combining differential ultracentrifugation, precipitation, and filtration methods [151]. For enhanced storage after isolation, techniques like cryopreservation [152] and lyophilization [153] are being refined with cryoprotectants to maintain exosome integrity for administrations in clinical trials according to needs and limitations in practices [146]. Efforts should also be made to standardize these processes in accordance with Good Manufacturing Practices (GMP), ensuring product consistency, safety, and efficacy. This includes exploring non-invasive delivery methods such as oral administration, which could significantly enhance patient compliance [154]. Future work on exosomal IRI therapies may have to work on optimizing biomanufacturing process for scaled and reliable exosome production.

#### Exploration of new sources of exosomes

Exploring new sources of therapeutic exosomes may reveal variants with superior or unique therapeutic characteristics, expanding the possibilities for treating a range of IRI. As previously stated, urine-derived stem cells stand out as a promising source of exosomes, possessing the inherent benefits of accessibility and relatively free of ethical concerns compared to other human cells [133]. Animal-derived exosomes, particularly from milk, have been shown to offer unique therapeutic potentials and larger production capacities, although a more thorough analysis of cross-activity with humans should be required [155]. Also, exosomes derived from plants have shown anti-inflammatory and antioxidant effects in mouse gut host cells [156, 157]. Future studies on exosomal IRI therapies can also explore these sources in both animal and plant models, broadening the therapeutic options.

## Data Availability

Data sharing is not applicable to this article as no datasets were generated or analyzed during the current study.
